# Supplementary material for: Exploratory Evaluation of Topical Tacrolimus for Prevention of Breast Cancer-Related Arm Lymphedema: A Multicenter Non-Randomized Pilot Study
Source: Cancers (Basel). 2025 Nov 24;17(23):3753. doi: 10.3390/cancers17233753 (PMC12691113; doi:10.3390/cancers17233753)
Supplement: Supplementary file 1 [file cancers-17-03753-s001.zip › cancers-3958948-supplementary.pdf]

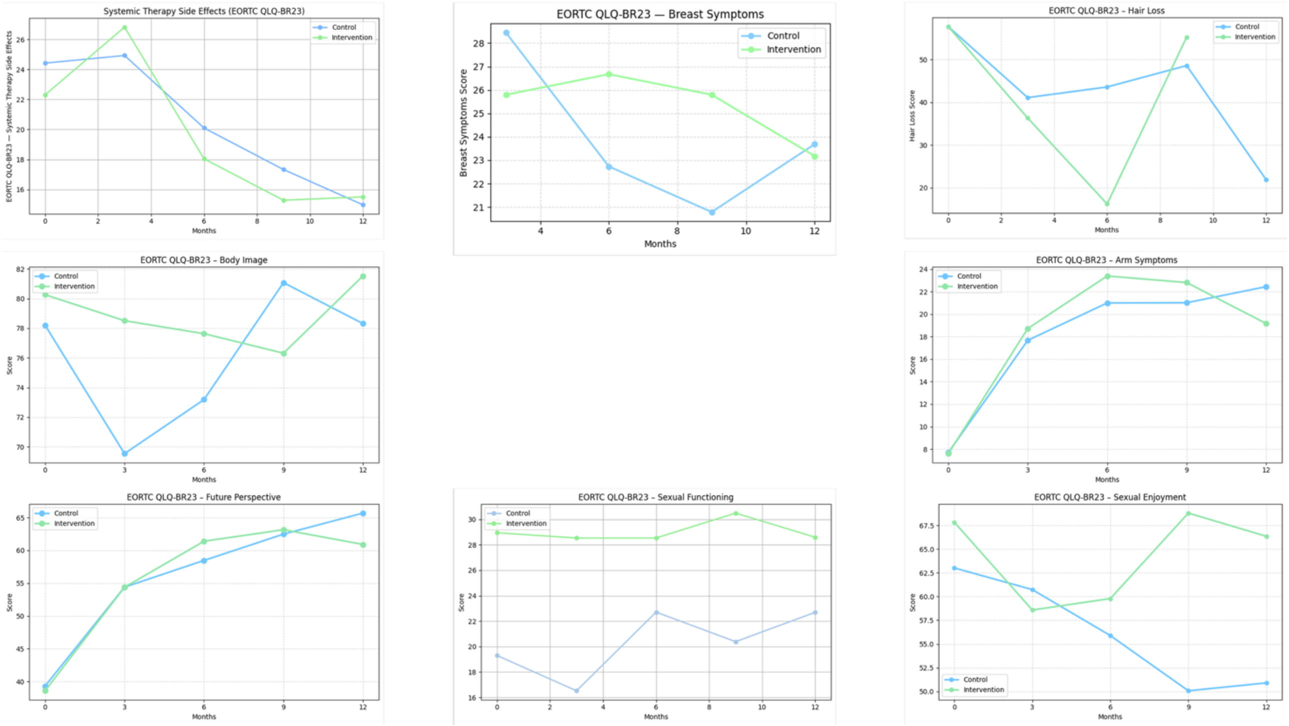

Supplemental Figure S1. EORTC QLQ-BR23 Domain Scores. This figure displays the trajectories of eight EORTC QLQ-BR23 domains over the 12-month follow-up period for the control group (blue) and intervention group (green). Each panel represents a separate domain: systemic therapy side effects, breast symptoms, hair loss, body image, arm symptoms, future perspective, sexual functioning, and sexual enjoyment. Scores are presented at baseline and at 3-, 6-, 9-, and 12-month follow-ups.
